# Therapeutic targeting of the host-microbiota-immune axis: implications for precision health

**DOI:** 10.3389/fimmu.2025.1570233

**Published:** 2025-04-29

**Authors:** Asiya Nazir, Fathima Hasnain Nadeem Hussain, Tuahir Hassan Nadeem Hussain, Rania Al Dweik, Afsheen Raza

**Affiliations:** ^1^ Department of Biomedical Sciences, College of Health Sciences, Abu Dhabi University, Abu Dhabi, United Arab Emirates; ^2^ College of Medicine and Health Sciences, Khalifa University, Abu Dhabi, United Arab Emirates

**Keywords:** gut microbiota, microbiota-immune interactions, microbiota therapeutics, probiotics, fecal microbiota transplantation, precision medicine and microbiome, microbial dysbiosis

## Abstract

The human body functions as a complex ecosystem, hosting trillions of microbes that collectively form the microbiome, pivotal in immune system regulation. The host-microbe immunological axis maintains homeostasis and influences key physiological processes, including metabolism, epithelial integrity, and neural function. Recent advancements in microbiome-based therapeutics, including probiotics, prebiotics and fecal microbiota transplantation, offer promising strategies for immune modulation. Microbial therapies leveraging microbial metabolites and engineered bacterial consortia are emerging as novel therapeutic strategies. However, significant challenges remain, including individual microbiome variability, the complexity of host-microbe interactions, and the need for precise mechanistic insights. This review comprehensively examines the host microbiota immunological interactions, elucidating its mechanisms, therapeutic potential, and the future directions of microbiome-based immunomodulation in human health. It will also critically evaluate challenges, limitations, and future directions for microbiome-based precision medicine.

## 1 Introduction

The human body is a complex ecosystem, housing trillions of microbes, including bacteria, viruses, fungi, and archaea, that collectively form the human microbiome (1). These microbial communities, residing predominantly in the gastrointestinal tract but also on the skin, respiratory tract (2, 3), and other mucosal surfaces, have evolved in a highly regulated relationship with the host immune system. This dynamic interplay, referred to as the host-microbe immunological axis, is essential for maintaining homeostasis, influencing not only immune system development and function but also a broad spectrum of physiological processes, including metabolism, epithelial integrity, and neural function. Disruptions to this delicate balance have been implicated in the pathogenesis of a range of diseases, from autoimmune disorders to metabolic syndromes and even neurodegenerative diseases. Recent advances in microbiome research have underscored the importance of microbial composition and diversity in shaping host immune responses. It is now well-established that the gut microbiota is a central player in modulating innate and adaptive immunity (4). The host immune system, in turn, regulates microbial composition, creating a bidirectional axis that is fundamental for health. Dysbiosis, or an imbalance in the microbial community, can trigger immune dysregulation, leading to chronic inflammation and disease pathogenesis. The immune system’s recognition of microbial signals through pattern recognition receptors (PRRs), such as Toll-like receptors (TLRs), and its capacity to generate appropriate immune responses to microbeshave emerged as central themes in the study of host-microbe interactions (5, 6). In this context, the modulation of the host-microbe immunological axis presents a promising therapeutic approach for a wide range of human health conditions. Therapeutic strategies aimed at restoring or maintaining microbial balance (7), such as probiotics, prebiotics, dietary interventions, and fecal microbiota transplantation (FMT) (8, 9) have shown potential in the management of diseases such as inflammatory bowel disease (IBD), allergies, and even metabolic disorders (10). More recently, the exploration of microbiome-based immunotherapies has gained traction, with microbial products or metabolites being considered as potential modulators of immune responses (8). Similarly, in obesity, an altered gut microbiome composition has been linked to increased energy harvest, systemic inflammation, and metabolic dysregulation, contributing to insulin resistance and other comorbidities (11). Understanding dysbiosis and its role in these conditions is crucial for developing microbiome-targeted therapeutic strategies. These interventions hold the promise of targeted treatments that address the symptoms of diseases and their underlying immunological causes (12). However, while the potential for modulating the host-microbe immunological axis for therapeutic benefit is vast, significant challenges remain. The heterogeneity of microbiome composition across individuals, the complex nature of host-microbe interactions, and the potential for unintended consequences complicate the development of standardized, effective therapies (13). Moreover, a deeper understanding of the mechanisms by which microbes influence the immune system ranging from immune cell differentiation and activation to cytokine production and antigen presentation, is still needed (14–18). Additionally, the role of the microbiome in regulating immune responses in tissues beyond the gut, including the skin, lungs (19), and brain, is an area of intense research with significant implications for systemic health (12). This review aims to provide a comprehensive overview of the current state of knowledge on the modulation of the host-microbe immunological axis in human health. It explored the bidirectional relationship between the immune system and the microbiota, focusing on the mechanisms by which microbes influenced immune responses and how these interactions were modulated for therapeutic benefit. The review also examined emerging therapeutic strategies that targeted microbiome (20) and immune system, including probiotic and prebiotic interventions, microbiome-based drug development, and the potential use of microbiota-derived metabolites in immune modulation. Finally, the review will address the challenges and future directions in this rapidly evolving field, providing insight into the potential for microbiome-based immunomodulation in treating and preventing immune-related diseases.

Precision medicine in microbiome-based therapies involves tailoring interventions to an individual’s unique microbial composition, genetic background, and immune response (20). By leveraging advanced sequencing technologies and computational tools, precision medicine aims to develop targeted microbial interventions, including probiotics, prebiotics, and microbiota-derived metabolites, to modulate the immune system effectively (21). This personalized approach enhances treatment efficacy, minimizes adverse effects, and holds promise for managing a range of immune-related disorders, from inflammatory diseases to cancer immunotherapy (22, 23).

## 2 Technological advances in understanding the immunological axis

Recent technological advancements, particularly high-throughput sequencing technologies, have significantly improved our ability to study microbiota-immune system interactions. Metagenomic, metatranscriptomic, and metabolomic techniques have provided valuable insights into the functional capabilities of microbial communities, including their ability to produce bioactive metabolites that can influence host immune responses (7). Additionally, single-cell RNA sequencing (scRNA-seq) has enabled the detailed examination of immune cell heterogeneity and the identification of specific immune cell subsets that interact with the microbiome, offering a deeper understanding of the cellular dynamics involved in immune modulation. Moreover, advances in high-resolution imaging techniques, such as flow cytometry and multiplex immunofluorescence, have facilitated the real-time analysis of immune cell activation, migration, and cytokine production in response to microbial stimuli (**Table 1**). These technologies along with germ-free and gnotobiotic animal models have provided critical insights into the causal relationships between microbiome composition and immune system function (32). Furthermore, the development of organ-on-a-chip models and 3D tissue cultures has allowed for studying host-microbe interactions in more physiologically relevant contexts, bridging the gap between *in vitro* and *in vivo* models. These technological breakthroughs have significantly enhanced our ability to investigate the dynamic nature of the host-microbe immunological axis. They are paving the way for more targeted, personalized therapeutic interventions aimed at modulating the microbiome for immune modulation (19).

**Table 1:** Technological advances in deciphering the host-microbe immunological axis.

Technological Approach	Description	Key Insights	Strengths	Limitations	References
Metagenomics	Sequencing microbial genomes to assess functional potential.	Revealed SCFA production (e.g., butyrate) crucial for Treg differentiation and epithelial integrity.	Provides a broad view of microbial community composition and function.	Limited strain-level resolution due to reliance on reference databases.	(24, 25)
Metabolomics	Analyzing microbial metabolites and linking them to host immune pathways.	Identified metabolites (e.g., indoles) activating AhR to modulate inflammation.	Directly connects microbiome function to immune responses.	Attribution of metabolites to specific taxa remains challenging.	(26)
Transcriptomics	Captures changes in host gene expression during microbial interactions.	Elucidated TLR-mediated pathways involved in gut immune regulation.	Provides dynamic insights into host-microbe signaling.	Limited temporal resolution without longitudinal studies.	(27)
Single-Cell RNA Sequencing	High-resolution analysis of immune cell subsets and responses.	Identified distinct immune cell plasticity in conditions like IBD.	Offers unparalleled resolution of immune cell heterogeneity.	Loses spatial context of immune interactions within tissues.	(28)
Spatial Transcriptomics	Maps immune and microbial interactions within intact tissues.	Revealed disrupted cytokine gradients in IBD tissue regions with microbial dysbiosis.	Retains spatial context, enabling localized analysis of host-microbe interactions.	Resolution remains suboptimal, and data integration is computationally demanding.	(29)
Machine Learning (ML)	AI algorithms for integrating complex datasets and predicting patterns in immune responses.	Identified microbiome-based biomarkers and predicted immune-modulating microbial metabolites.	Enables large-scale data integration, biomarker discovery, and therapeutic design.	Often functions as a “black box,” with limited biological explainability.	(30, 31)
Reinforcement Learning (AI)	Optimizes synthetic microbiota for desired immunological effects.	Designed microbial consortia for anti-inflammatory responses and precision therapies.	Supports personalized medicine through patient stratification.	Requires extensive preclinical and clinical validation to ensure efficacy.	(31)

## 3 Mechanisms of communication between microbiota and the immune system

**Figure 1:**
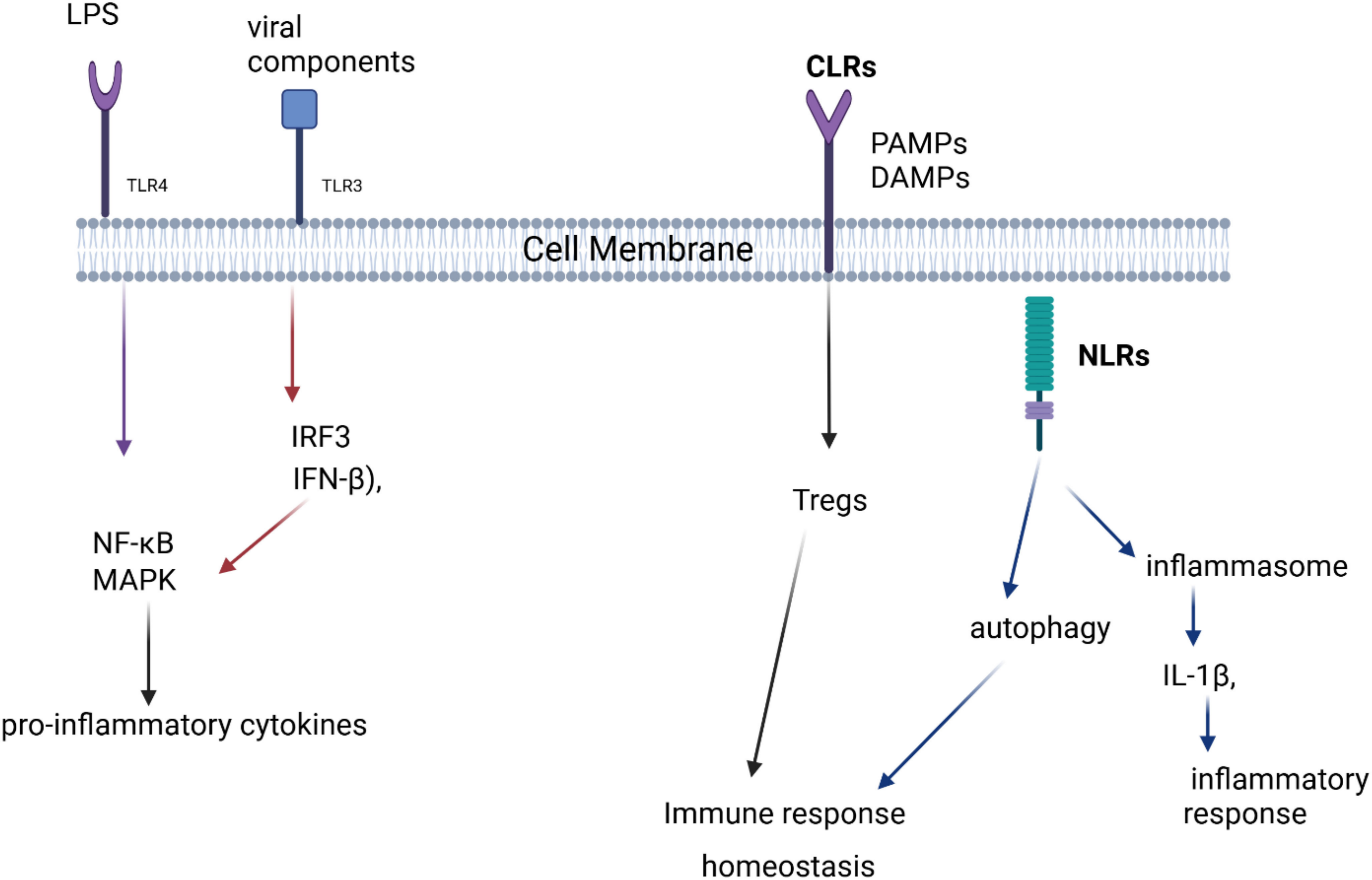
Pattern Recognition Receptors (PRRs) and Immune Signaling Pathways. This figure depicts how different pattern recognition receptors (TLRs, CLRs, and NLRs) recognize microbial components, leading to immune responses. TLR4 and TLR3 activate pro-inflammatory signaling via NF-κB, MAPK, and IFN-β, while CLRs contribute to immune homeostasis through Tregs. NLRs regulate autophagy and inflammasome activation, influencing inflammatory responses.

The communication between microbiota and the immune system is mediated through microbial metabolites, pattern recognition receptors (PRRs), and immune signaling pathways **Figure 1**. These interactions play a crucial role in maintaining immune homeostasis and protecting the host from pathogens.

### 3.1 Pattern recognition receptors

Pattern recognition receptors (PRRs) are a crucial component of the immune system that enables the host to detect and respond to microbial invaders and danger signals. These receptors are found on various immune cells and are responsible for recognizing conserved molecular structures called pathogen-associated molecular patterns (PAMPs) and damage-associated molecular patterns (DAMPs). The recognition of these patterns initiates immune responses that are essential for maintaining immune homeostasis and protecting the host from infections, tissue damage, and diseases such as cancer. PRRs are classified into several types, each specialized for recognizing different microbial or host-derived patterns. The main categories of PRRs include Toll-like receptors (TLRs), NOD-like receptors (NLRs), C-type lectin receptors (CLRs), and the various PAMPs and DAMPs recognized by these receptors (33, 34).

#### 3.1.1 Toll-like receptors

TLRs, a subset of PRRs, are transmembrane receptors primarily expressed on immune cells such as dendritic cells, macrophages, and neutrophils. Each TLR specializes in recognizing distinct microbial signatures, for example, TLR4 detects lipopolysaccharide (LPS) from Gram-negative bacteria, while TLR3 recognizes double-stranded RNA from viruses (35, 36). Upon ligand binding, TLRs activate intracellular signaling pathways that stimulate pro-inflammatory cytokine secretion, recruit immune cells, and initiate adaptive immune responses (37).

#### 3.1.2 NOD-like receptors

NLRs are intracellular PRRs that detect bacterial PAMPs, such as peptidoglycans, and activate the inflammasome, which processes cytokines such as IL-1β (38). NLR activation is crucial for immune system homeostasis, but dysregulation can lead to chronic inflammatory conditions (39). Certain NLRs also regulate autophagy, aiding intracellular pathogen clearance and maintaining cellular balance (40).

Some NLRs also detect non-microbial danger signals and form large cytoplasmic structures known as inflammasomes. These inflammasomes link the detection of microbial products and metabolic stress to the activation of proinflammatory cytokines such as IL-1beta and IL-18. The NALP3 complex has been implicated in several autoinflammatory diseases, including gout (41).

#### 3.1.3 C-type lectin receptors

CLRs specialize in recognizing carbohydrate structures on pathogens, primarily targeting fungi, bacteria, and viruses. They are essential for immune activation, antigen uptake, and pathogen recognition, particularly in fungal infections (42, 43).

CLRs are involved in modulating immune responses to fungi, bacteria, and viruses, and play a role in pathogen recognition, antigen uptake, and immune activation (44). CLRs also influence T-helper cell polarization and regulatory T cell (Treg) development, promoting immune homeostasis (45).

#### 3.1.4 PAMPs and DAMPs

PRRs recognize both pathogen-associated molecular patterns (PAMPs) and damage-associated molecular patterns (DAMPs) to differentiate between infection and tissue damage (46). DAMPs, such as high-mobility group box 1 (HMGB1), are released during cellular injury and can trigger inflammation via receptors like TLR4 (47). This dual recognition system balances pathogen clearance with tissue repair (48).

### 3.2 Short-chain fatty acids

**Figure 2:**
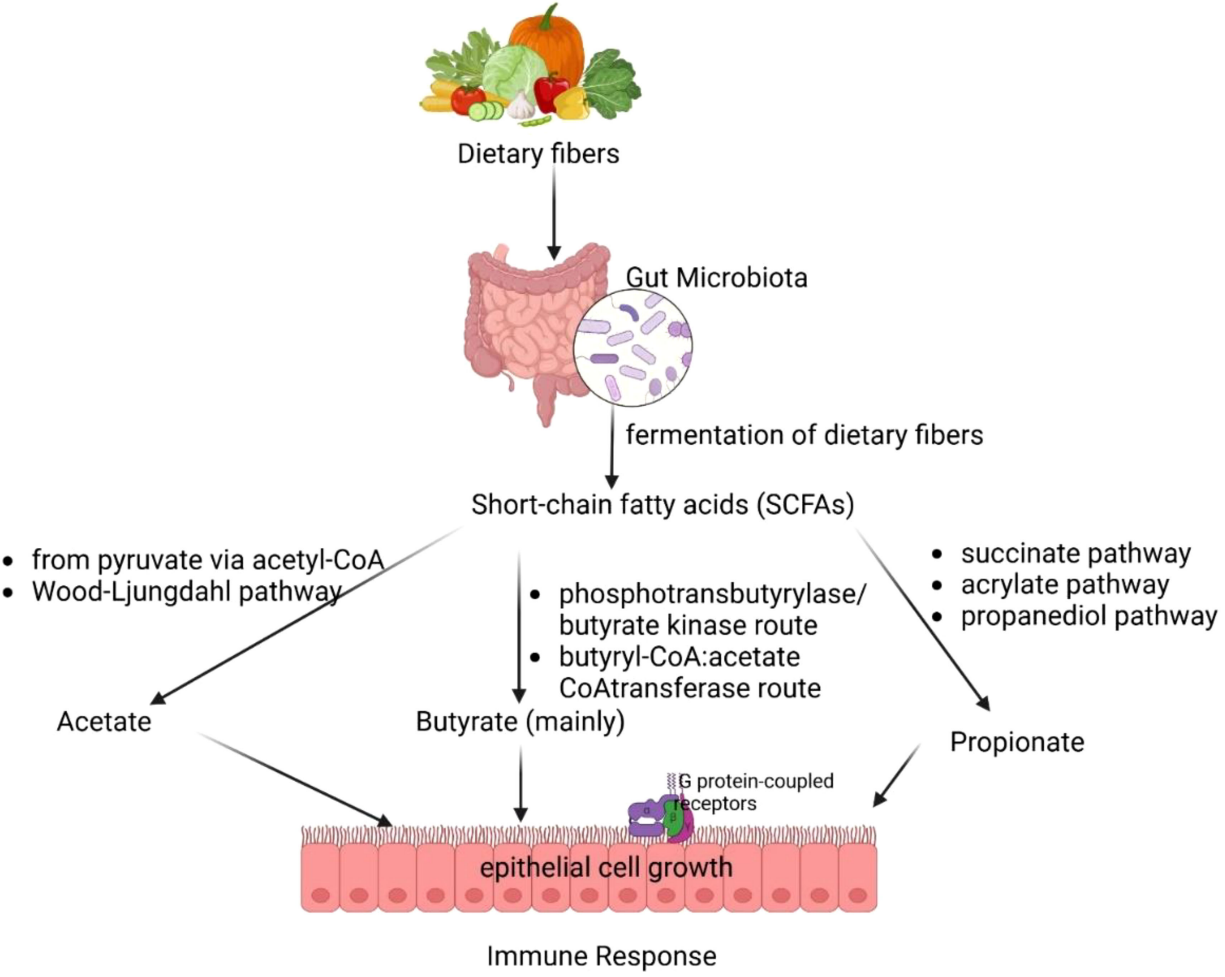
Gut microbial fermentation of dietary fibers and short-chain fatty acid production.

The gut microbiota’s production of SCFAs, including acetate, propionate, and butyrate, plays a fundamental role in maintaining immune tolerance, gut health, and host metabolism (49, 50). **Figure 2** illustrates how gut microbiota ferments dietary fibers to produce SCFAs, including acetate, butyrate, and propionate. These SCFAs play a crucial role in epithelial cell growth, immune response, and metabolic regulation. These microbial metabolites are primarily produced in the colon through distinct bacterial pathways. Acetate is synthesized via the pyruvate pathway by *Akkermansia muciniphila*, *Bacteroides* spp., *Bifidobacterium* spp., and *Ruminococcus* spp. (51, 52). Propionate is generated through the succinate, acrylate, and propanediol pathways by species such as *Bacteroides* spp., *Veillonella* spp., and *Roseburia inulinivorans* (53). Butyrate a key immunomodulatory SCFA, is predominantly via the phosphotransbutyrylase/butyrate kinase and butyryl-CoA:acetate CoA-transferase pathwaysinvolving *Coprococcus catus*, *Faecalibacterium prausnitzii*, and *Eubacterium rectale* (54, 55).

Among butyrate-producing bacteria, *Faecalibacterium prausnitzii* plays a crucial role due to its ability to modulate gut immune responses and reinforce epithelial integrity (55). This bacterium exerts anti-inflammatory effects by inhibiting NF-κB activation and stimulating IL-10 production, thereby reducing gut inflammation and maintaining homeostasis (56). Additionally, butyrate enhances the expression of tight junction proteins such as ZO-1 and occludin, essential for maintaining gut barrier function (57). Disruptions in *F. prausnitzii* abundance have been linked to inflammatory bowel disease (IBD) and colorectal cancer, emphasizing its potential as a probiotic candidate for microbiota-targeted therapies (58).

SCFAs exert systemic effects by modulating immune function and metabolism. Acetate, the most abundant SCFA in circulation, can cross the blood-brain barrier to regulate appetite via central homeostatic mechanisms (59). Propionate, predominantly metabolized in the liver, contributes to lipid and glucose metabolism but remains at low peripheral concentrations (50). Butyrate plays a key role in reinforcing gut barrier integrity, reducing inflammation, and promoting regulatory T cell (Treg) differentiation, which is crucial for immune tolerance and the suppression of excessive inflammation (60–62). It also directly modulates dendritic cells and macrophages, enhancing their ability to recognize pathogen-associated molecular patterns (PAMPs) and regulating pro-inflammatory cytokine secretion (63).

A significant mechanism underlying butyrate’s immunomodulatory effects is its inhibition of histone deacetylases (HDACs), leading to epigenetic modifications that regulate gene expression associated with immune homeostasis (64–66). This process fosters anti-inflammatory signaling, supports tissue repair, and balances immune responses in conditions such as infections and cancer. Mammals rely on gut bacteria to break down indigestible dietary components, such as fiber, generating SCFAs as metabolic byproducts that contribute to host health and disease prevention (11). The dynamic interplay between diet, microbial metabolism, and immune regulation highlights the potential of SCFAs as therapeutic targets for inflammatory and metabolic disorders.

### 3.3 Cytokine signaling pathways

Cytokine signaling pathways are fundamental to immune regulation, with the microbiome playing a key role in modulating both pro-inflammatory and anti-inflammatory responses. Pro-inflammatory cytokines, such as TNF-α, IL-1β, IL-6, and IFN-γ, are critical in initiating inflammatory reactions and serve as primary mediators of immune responses during infection or injury (67). Microbial components, including lipopolysaccharides (LPS), activate signaling pathways such as the nuclear factor NF-κB pathway through Toll-like receptors, particularly TLR4, TLR3 and TLR5. The recognition of LPS by TLR4 triggers NF-κB activation, activator protein-1 (AP-1), and mitogen-activated protein kinase (MAPK) (68). These pathways stimulate the release of proinflammatory cytokines and chemokines, including interleukin (IL)-1, tumor necrosis factor (TNF)-α, and IL-6 (44, 69, 70). Cytokines are versatile proteins that play a key role in regulating osteoclast development and bone resorption, altering vascular endothelial permeability, and attracting immune cells to sites of inflammation (44, 71). The recognition of viral components by TLR3 triggers the TRIF-mediated signaling pathway, leading to the activation of Interferon Regulatory Factor 3 (IRF3) and the subsequent production of Interferon Beta (IFN-β), which plays a crucial role in inhibiting viral replication (72). Additionally, TLR3 stimulation activates the MAP kinase and NF-κB signaling pathways, promoting the production of pro-inflammatory cytokines (73).

This cytokine signaling process involves a highly regulated molecular cascade that is crucial for maintaining immunological homeostasis. Immune cells, such as dendritic cells and macrophages, recognize PAMPs from microbes or host-derived signals, such as SCFAs produced by microbiota. Upon recognition, these cells release cytokines, which can either promote or suppress inflammation, depending on the signals received (66). Cytokines bind to specific receptors on target cells, initiating intracellular signaling pathways involving kinases and transcription factors like NF-κB or STAT proteins. Once activated, these transcription factors translocate to the nucleus and regulate the expression of genes critical for immune responses, thereby enhancing T and B cell functions to combat infections and malignancies (74).

For instance, in inflammatory bowel disease (IBD), an overactive cytokine response leads to chronic inflammation in the gut, driven by excessive production of TNF-α, IL-1β, and IL-6, which contribute to tissue damage and immune dysregulation (75). Similarly, in rheumatoid arthritis, dysregulated cytokine signaling—particularly involving TNF-α and IL-6—results in persistent joint inflammation and autoimmunity (76). In contrast, in cancer, certain cytokines such as IL-10 and TGF-β suppress immune responses, allowing tumor cells to evade immune detection and promote tumor progression (77). These disease models illustrate the dual role of cytokines in either driving or suppressing immune responses, emphasizing the necessity of maintaining a precise balance between pro-inflammatory and anti-inflammatory signals.

To prevent excessive immune activation and inflammation, the immune system carefully balances pro-inflammatory and anti-inflammatory signals, ensuring a controlled response that maintains systemic homeostasis while addressing challenges such as infections, cancer, and interactions with the microbiota (34).

## 4 Distinction between symbiotic and pathogenic interactions

Host-microbe interactions can be broadly classified into symbiotic and pathogenic relationships, depending on their immunological effects and the impact on host health. While pathogenic interactions often result in infection and disease, symbiotic relationships include both mutualistic and commensal associations, which help maintain host homeostasis and immune balance.

### 4.1 Pathogenic interactions: disrupting host integrity

Pathogenic interactions occur when microorganisms invade the host, triggering an immune response that aims to eliminate the threat but can also lead to inflammation and tissue damage. Pro-inflammatory cytokines like IL-6, TNF-α, and IFN-γ are released to recruit immune cells and amplify the response (78). Pathogens are detected by pattern recognition receptors (PRRs) such as TLRs and NLRs, which recognize microbial-associated molecular patterns and initiate immune signaling (79). The adaptive immune system further engages by activating effector T cells and stimulating antibody production (2). However, excessive immune activation can lead to collateral damage and worsen disease outcomes (80). For example, *Salmonella enterica* triggers IL-6-mediated gut inflammation (81), *Mycobacterium tuberculosis* evades immune detection while inducing chronic inflammation (82), and SARS-CoV-2 provokes a severe cytokine storm, causing lung injury and systemic complications (83).

### 4.2 Symbiotic interactions: a balanced coexistence

Symbiotic microbes establish stable, non-harmful relationships with the host, offering benefits. These interactions can be mutualistic, where both the host and microbe benefit or commensal, where the microbe benefits without harming the host (84). In mutualistic interactions, microbes regulate immune responses, enhance barrier protection, and support immune homeostasis. For instance, Bacteroides fragilis produces polysaccharide A (PSA), which promotes regulatory T cells and prevents colitis (85). *Lactobacillus reuteri* secretes antimicrobial peptides that help protect the mucosal lining and prevent pathogen colonization (86). Additionally, mutualistic microbes stimulate anti-inflammatory cytokines like IL-10 and TGF-β, promoting immune tolerance and preventing excessive inflammation (87). They also strengthen epithelial integrity, as seen with *Lactobacillu*s and *Bifidobacterium*, and foster immune balance through regulatory T cell activity (2).

### 4.3 Commensal relationships: coexistence without harm

In contrast, commensal relationships, where microbes coexist with the host without causing harm, are typically associated with a more regulated immune response. The immune system often promotes the production of anti-inflammatory cytokines, such as IL-10, and enhances processes supporting immune tolerance, including the activity of regulatory T cells (Tregs). For example, *Staphylococcus epidermidis* on the skin produces antimicrobial molecules that protect against *Staphylococcus aureus* infections, preventing harmful overgrowth (88). Similarly, the gut microbiome maintains a balance between beneficial and opportunistic bacteria, reducing the risk of infections like *Clostridioides difficile* (89). These mechanisms work to prevent unnecessary immune activation and inflammation, allowing commensals to persist in a balanced state without triggering excessive inflammatory responses (87). This delicate equilibrium between immune activation and regulation is crucial for maintaining host homeostasis while permitting the coexistence of beneficial microorganisms without compromising immune system integrity (90, 91).

## 5 Immunological changes during various illness

Immunological alterations during disease progression vary significantly based on factors such as the nature of the disease, its underlying etiology, and the extent to which it influences immune function. These changes can be categorized into two primary components: innate immunity, which provides the body’s initial, rapid defense against pathogens, and adaptive immunity, which generates a more specific, durable immune response. The interplay between these systems can lead to the activation, suppression, or dysregulation of immune responses, depending on the pathophysiological context of the illness (**Table 2**). Dysregulation may occur when the immune system’s normal functioning is disrupted, resulting in inadequate responses, excessive inflammation, or autoimmunity, thus exacerbating the disease and complicating recovery (92).

**Table 2:** Role of Short-chain fatty acids (SCFAs) and Indole derivates in human health.

	SCFAs	Indole derivatives and tryptophan metabolism
Mechanism	• Produced when the gut bacteria ferments dietary fibers • Preserves the gut barrier • Activates T cells • Suppresses pro-inflammatory cytokines like TNF-α.	• Activates AhR • Stimulates Tregs • Help gut bacteria convert tryptophan into indole derivatives.
Benefits	• Boosts immunological and metabolic function • Decreases chronic inflammation • Improves gut health.	• Regulates inflammation • Maintains immunological balance • Strengthen the gut barrier • Boosts mucosal immunity

### 5.1 Immunological targets for better health

Immunological interventions aimed at promoting health encompass enhancing immune function, preventing infections, and managing chronic diseases. Targeting cytokines such as TNF-α and IL-6 has been effective in reducing inflammation in autoimmune disorders like rheumatoid arthritis. Likewise, immune checkpoint inhibitors targeting PD-1, PD-L1, and CTLA-4 have revolutionized cancer therapy by reactivating T cells to combat malignant cells. Another significant target is regulatory T cells (Tregs), which can be modulated to restore immune tolerance in autoimmune diseases or to enhance anti-tumor immunity (93). The gut microbiota plays a crucial role in immune modulation, with probiotics (94), prebiotics, and dietary interventions demonstrating therapeutic potential for conditions such as inflammatory bowel disease and obesity (95). Nutritional strategies (96), including supplementation with vitamin D, zinc, and omega-3 fatty acids, are also critical for maintaining optimal immune function (97). Additionally, targeting inflammasomes which are the key components responsible for the production of pro-inflammatory cytokines like IL-1β and IL-18—holds promise for treating diseases such as gout and autoimmune conditions (98). Vaccination remains a cornerstone of public health, offering cost-effective protection against infectious diseases and enhancing immunological memory. Technological advances, particularly mRNA vaccine platforms (99), have significantly improved immunity against pathogens such as influenza, HPV, and COVID-19 (100, 101). Toll-like receptors essential in pathogen recognition, are also being explored for their potential to boost immune responses against infections and cancers while mitigating excessive inflammation in autoimmune diseases (102, 103). Monoclonal antibodies and Janus kinase (JAK) inhibitors represent advanced therapeutic modalities for treating autoimmune disorders, cancers, and chronic inflammatory diseases by specifically targeting immune components. Furthermore, enhancing immune processes like antibody-dependent cellular cytotoxicity (ADCC) is being investigated for its potential to treat cancer and infections (104). By strategically modulating these immunological targets, it is possible to enhance immune function, improve disease management, and promote overall health (105). By strategically modulating these immunological targets, it is possible to enhance immune function, improve disease management, and promote overall health (106). Notably, microbiota-driven interventions hold immense promise in shaping immune responses by influencing T cell differentiation, mucosal immunity, and systemic inflammation. Harnessing microbial-derived metabolites, such as SCFAs, and engineered probiotics provides novel avenues for immunotherapy and personalized medicine (23). Advancing research in microbiota-immune interactions could lead to breakthroughs in disease prevention and therapeutic strategies, reinforcing the pivotal role of the gut microbiome in sustaining immune balance and health (107).

### 5.2 Immunotherapy targets

Monoclonal antibodies are sophisticated biological therapeutics that target specific antigens, thereby inducing their destruction or facilitating their elimination through mechanisms such as antibody-dependent cellular cytotoxicity (ADCC) and complement activation. These agents have demonstrated efficacy in reducing inflammation and joint damage in rheumatoid arthritis by neutralizing TNF-α and alleviating the inflammatory symptoms of psoriasis by targeting IL-17 and IL-23 (105). In oncology, monoclonal antibodies that block the PD-1/PD-L1 interaction restore T-cell functionality in non-small cell lung cancer, enhancing the immune system’s capacity to target and eliminate tumors. Similarly, anti-CD20 monoclonal antibodies in hematological malignancies promote B cell depletion through ADCC and complement activation. In the context of inflammatory bowel disease integrin α4β7 inhibitors limit the migration of lymphocytes to the gut mucosa, thereby reducing intestinal inflammation (108). In parallel, JAK inhibitors, which are small molecule agents, interfere with the JAK-STAT signaling pathway which is a central regulator of pro-inflammatory cytokines and cellular growth factors. By inhibiting specific JAK isoforms such as JAK1, JAK2, and JAK3, these inhibitors reduce cytokine signaling and inflammation (109). For instance, JAK1 and JAK3 inhibitors are effective in managing cytokine-driven inflammatory responses in conditions like rheumatoid arthritis and ulcerative colitis. Targeting JAK2 specifically addresses abnormal cytokine signaling and mitigates tissue fibrosis in myelofibrosis. Additionally, JAK inhibitors have been shown to reduce skin inflammation in atopic dermatitis by modulating dysregulated cytokine pathways. In the context of graft-versus-host disease (GVHD), these inhibitors play a critical role in limiting immune-mediated tissue damage, thus providing essential immunological control following transplantation (**Table 3**) (115). Collectively, these therapeutic agents, both monoclonal antibodies and JAK inhibitors, represent significant advances in the treatment of a variety of inflammatory and immune-mediated disorders, leveraging distinct but complementary mechanisms of action to modulate immune system activity and reduce pathological inflammation.

**Table 3:** Immunological axis in the onset of various physiological and metabolic diseases.

	Feature	Immune mechanism	References
Cancer	Tumors create immunosuppressive microenvironments, evading immune detection through reduced antigen presentation or inhibitory molecules like PD-L1.	Tumors restrict T cell activation, whereas immune evasion limits identification and eradication.	(110)
Autoimmune Diseases	The immune system attacks self-cells and damages organs. Resulting from genetic, environmental, and regulatory disturbances. Central and peripheral tolerance inhibit self-reactivity.	Tolerance breakdown activates autoreactive cells. Pro-inflammatory cytokines contribute to inflammation. Cytokine treatments show potential.	111)
Metabolic and Chronic Diseases	Linked with immunological disorders. Diabetes: High blood sugar affects neutrophil activity and T cell responses, increasing the infection risk. Obesity causes low-grade inflammation from higher pro-inflammatory cytokines (IL-6, TNF-α).	Systemic inflammation speeds up the course of illness; obesity-related impaired cytokine control results in compromised immune function.	(112, 113)
Infectious Diseases	Pro-inflammatory cytokines (IL-6, TNF-α) are released by neutrophils and macrophages in response to bacterial infections. Interferons and cytotoxic reactions are brought on by viral infections. Immune exhaustion is brought on by persistent illnesses, such as HIV.	T cells and antibodies can be used to activate adaptive immunity against bacterial infections. Parasites: TH1, TH2, and TH17 pathways - Viral infections: cytotoxic cell activation and interferon production.	(34, 114)
Allergic Disorders	Excessive response of the immune system to harmless stimuli (like pollen)-Inflammation caused by mast cell histamine release. Exhibiting a TH2-skewed immunological response.	IgE-mediated mast cell activation. Eosinophilic inflammation and imbalanced TH2 responses are features of chronic allergy-related conditions.	(114)

## 6 Immunological axis modulation strategies: case studies and applications in each domain

Immunological axis modulation involves the regulation of the complex interactions between the immune system and microorganisms, such as gut microbiota, to promote health and prevent disease. This section highlights case studies and applications of various strategies in modulating immune responses, with a focus on probiotics, prebiotics, and other therapeutic approaches that influence immune homeostasis and inflammation.

### 6.1 Probiotics and prebiotics in immune modulation

Probiotics are live microorganisms that confer a health benefit on the host when administered in adequate amounts (**Table 4**). They are primarily found in fermented foods and supplements, and their therapeutic potential is linked to their ability to modulate immune responses, particularly in the gut (124). A prominent example is *Lactobacillus rhamnosus* GG, a probiotic strain that has been used in clinical trials for treating inflammatory bowel diseases (IBD), such as ulcerative colitis (116)*. L. rhamnosus* GG has been shown to enhance gut mucosal immunity by promoting anti-inflammatory cytokines like IL-10 and reducing pro-inflammatory cytokines such as TNF-α. Other probiotic strains, including *Lactobacillus* and *Bifidobacterium* species, are known for their immunomodulatory effects, promoting a balanced immune response by stimulating the production of regulatory T cells (Tregs) and strengthening mucosal immunity (117, 125). These probiotics also improve gut barrier function and reduce intestinal inflammation, helping prevent infections and alleviating the severity of autoimmune diseases and allergies (94). The beneficial effects of probiotics on immune regulation are also supported by their ability to increase gut microbiota diversity, which has been shown to help restore immune tolerance and mitigate disease pathology (118).

**Table 4:** Probiotics: composition, duration, target diseases, and efficacy.

Probiotic	Composition	Duration of Therapy	Target Diseases	Efficacy	Reference
*Lactobacillus rhamnosus* GG	Single strain probiotic	4–8 weeks	Inflammatory Bowel Disease (IBD), Diarrhea, Allergies	Reduces gut inflammation, strengthens mucosal immunity	(116)
*Bifidobacterium breve*	Single strain probiotic	6–12 weeks	Necrotizing Enterocolitis (NEC), Infant Colic	Enhances gut barrier function, reduces colic symptoms	(1)
*Saccharomyces boulardii*	Yeast probiotic	4–6 weeks	Antibiotic-associated diarrhea, Clostridioides difficile infection	Reduces diarrhea incidence, restores gut flora balance	(117)
*Lactobacillus acidophilus & Bifidobacterium bifidum*	Multi-strain probiotic	8–12 weeks	Irritable Bowel Syndrome (IBS), Atopic Dermatitis	Alleviates bloating, improves gut microbiota composition	(118)
*Streptococcus thermophilus*	Single strain probiotic	6–10 weeks	Lactose Intolerance, Immune modulation	Enhances lactose digestion, boosts gut immunity	(119)
*VSL#3*	Multi-strain probiotic blend (8 bacterial strains)	12+ weeks	Ulcerative Colitis, Pouchitis	Reduces inflammation, supports remission maintenance	(120)
*Escherichia coli Nissle 1917*	Single strain probiotic	6–12 weeks	Ulcerative Colitis, Irritable Bowel Syndrome	Supports gut homeostasis, reduces inflammation	(49)
*Lactobacillus reuteri*	Single strain probiotic	4–6 weeks	Infant Colic, H. pylori Infection	Reduces colic symptoms, inhibits pathogen growth	(121)
*Bacillus coagulans*	Spore-forming probiotic	6–8 weeks	Rheumatoid Arthritis, Gastrointestinal Infections	Reduces inflammation, enhances immune response	(122)
*Clostridium butyricum*	Butyrate-producing probiotic	8–12 weeks	Inflammatory Bowel Disease, Metabolic Disorders	Improves gut barrier integrity, modulates immune function	(123)

In addition to probiotics, prebiotics have gained significant attention as a tool for modulating immune responses (119). Prebiotics are non-digestible fibers that selectively stimulate the growth of beneficial gut bacteria (**Table 5**). These fibers influence immune function by fostering a gut microbiota composition that promotes immune tolerance, particularly in preventing and treating allergic diseases (126). One key case study involves the supplementation of inulin, a prebiotic, in infants at high risk for allergies. The intervention resulted in a reduced incidence of atopic dermatitis, along with an increase in beneficial bacteria like *Bifidobacterium* and *Lactobacillus*, which support immune regulation (49). Similarly, oligofructose supplementation in children with allergic rhinitis demonstrated a reduction in allergen-specific IgE production, which is linked to allergic responses, and improved the balance between pro-inflammatory Th2 cells and Tregs (122). This suggests that prebiotics can help alleviate allergic symptoms by modulating the immune system and fostering a more balanced, less inflammatory immune response.

**Table 5:** List of available prebiotics, their sources, mechanism of action, duration of therapy, target diseases, and efficacy.

Prebiotic	Source	Mechanism of Action	Duration of Therapy	Target Diseases	Efficacy
Inulin	Chicory root, bananas, onions	Stimulates *Bifidobacterium* growth	8–12 weeks	Atopic Dermatitis, Gut Dysbiosis	Reduces allergic symptoms and enhances gut microbiota composition (49)
Oligofructose	Wheat, garlic, asparagus	Modulates Th2/Treg balance	6–10 weeks	Allergic Rhinitis, Asthma	Reduces IgE-mediated responses and inflammation (121)
Galacto-oligosaccharides (GOS)	Dairy products, legumes	Promotes beneficial gut bacteria	4–8 weeks	Infant Gut Health, Immune Support	Enhances gut barrier integrity and immune function (126)
Resistant Starch	Whole grains, potatoes	Increases SCFA production	10–16 weeks	Type 2 Diabetes, Obesity	Enhances butyrate production and improves insulin sensitivity (127)
Arabinoxylan	Whole wheat, rice bran	Supports *Bacteroides* species	8–12 weeks	Gut Health, Metabolic Disorders	Improves microbial diversity and reduces inflammation (128)

**Figure 3:**
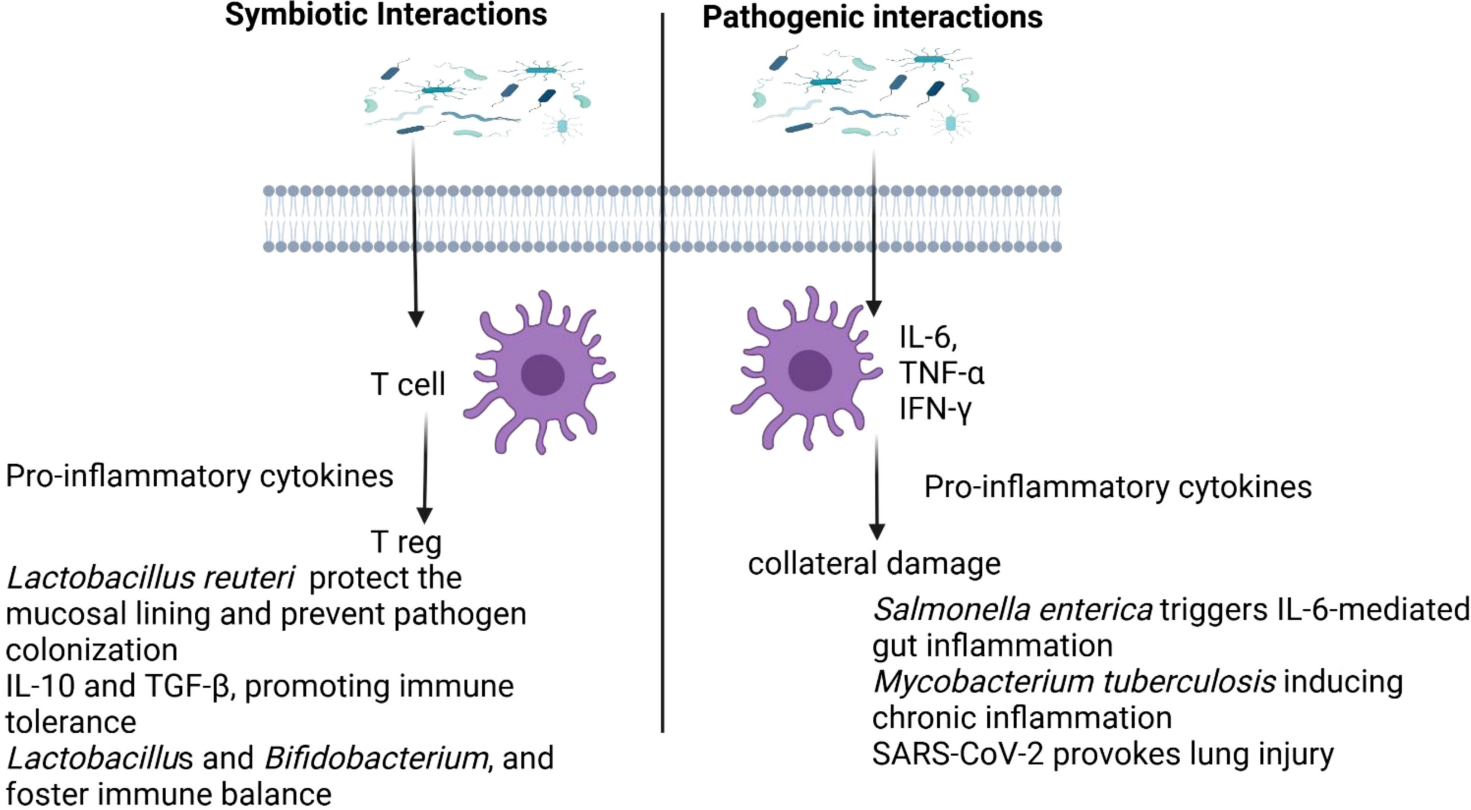
Differing impacts of symbiotic and pathogenic microbial interactions on immune regulation.

A key mediator of both probiotic and prebiotic effects on immune regulation is SCFAs, particularly butyrate (128). Butyrate plays a crucial role in maintaining intestinal barrier integrity, enhancing the regeneration of epithelial cells, and reducing intestinal permeability, which is often implicated in inflammatory diseases (129). Butyrate also activates Tregs, which are essential for immune tolerance, and inhibits the production of pro-inflammatory cytokines such as TNF-α, further promoting a balanced immune response (122). The production of SCFAs, including butyrate, is influenced by the gut microbiota’s fermentation of dietary fibers, underscoring the importance of a balanced microbiome in producing these metabolites (127). Beyond their gut health benefits, SCFAs like butyrate exert systemic effects, modulating immune responses and potentially protecting against chronic diseases, including obesity, type 2 diabetes, and autoimmune disorders (130, 131). **Figure 3**. illustrates the contrasting effects of symbiotic and pathogenic microbial interactions on the immune response

Together, probiotics and prebiotics work synergistically to enhance immune health by improving gut microbiota diversity, promoting the production of anti-inflammatory cytokines, and regulating immune responses through metabolites like butyrate. These effects are essential for reducing inflammation, preventing infections, and mitigating the risks associated with autoimmune diseases and allergies (94, 120).

### 6.2 Fecal microbiota transplantation

Fecal microbiota transplantation (FMT) has emerged as a revolutionary immunological strategy for modulating the host-microbe axis, particularly in cases of diseases where the gut microbiota is disrupted. FMT involves transferring fecal material from a healthy donor into the gastrointestinal tract of a recipient, thereby restoring microbial diversity and balance (132). One of the most well-documented and successful applications of FMT is in the treatment of recurrent *Clostridium difficile* (*C. difficile*) infection, which often arises after antibiotic treatments disrupt the natural gut microbiota. A landmark case study demonstrated that FMT achieved a 90% success rate in curing recurrent *C. difficile* infections (133). Patients who underwent FMT experienced significant restoration of gut microbiota diversity, which helped re-establish immune homeostasis. Safety and efficacy of fecal microbiota transplantation (FMT) as a modern adjuvant therapy in various diseases and disorders (134). The restored microbiota was able to suppress the overgrowth of *C. difficile* and reduced intestinal inflammation, offering a direct example of how microbiota modulation can enhance immune function and control infection (135). Beyond infectious diseases, FMT has shown promise in autoimmune and inflammatory disorders. For instance, in patients with ulcerative colitis (UC), a form of inflammatory bowel disease (IBD), FMT has been explored as a means of reducing inflammation and modulating the immune system (136). A study involving UC patients who received FMT showed improvements in clinical symptoms, including reduced disease activity and inflammation. This therapeutic effect was attributed to changes in the gut microbiota that promoted a shift from pro-inflammatory to anti-inflammatory immune responses, including an increase in regulatory T cells (Tregs) that are crucial for immune tolerance (137). Additionally, FMT has been investigated in the context of multiple sclerosis (MS), an autoimmune disease where the immune system attacks the central nervous system (138). Preliminary clinical studies in MS patients receiving FMT have shown promising results, with some patients experiencing reduced disease progression and fewer relapses, potentially due to changes in gut microbiota composition that impact the peripheral immune system (134). These case studies highlight FMT’s potential not only for treating infections but also for modulating the immune system in autoimmune and inflammatory diseases by restoring microbial diversity and re-establishing immune balance, suggesting a new frontier for therapeutic interventions in immunology.

### 6.3 Dietary interventions in inflammatory diseases

**Figure 4:**
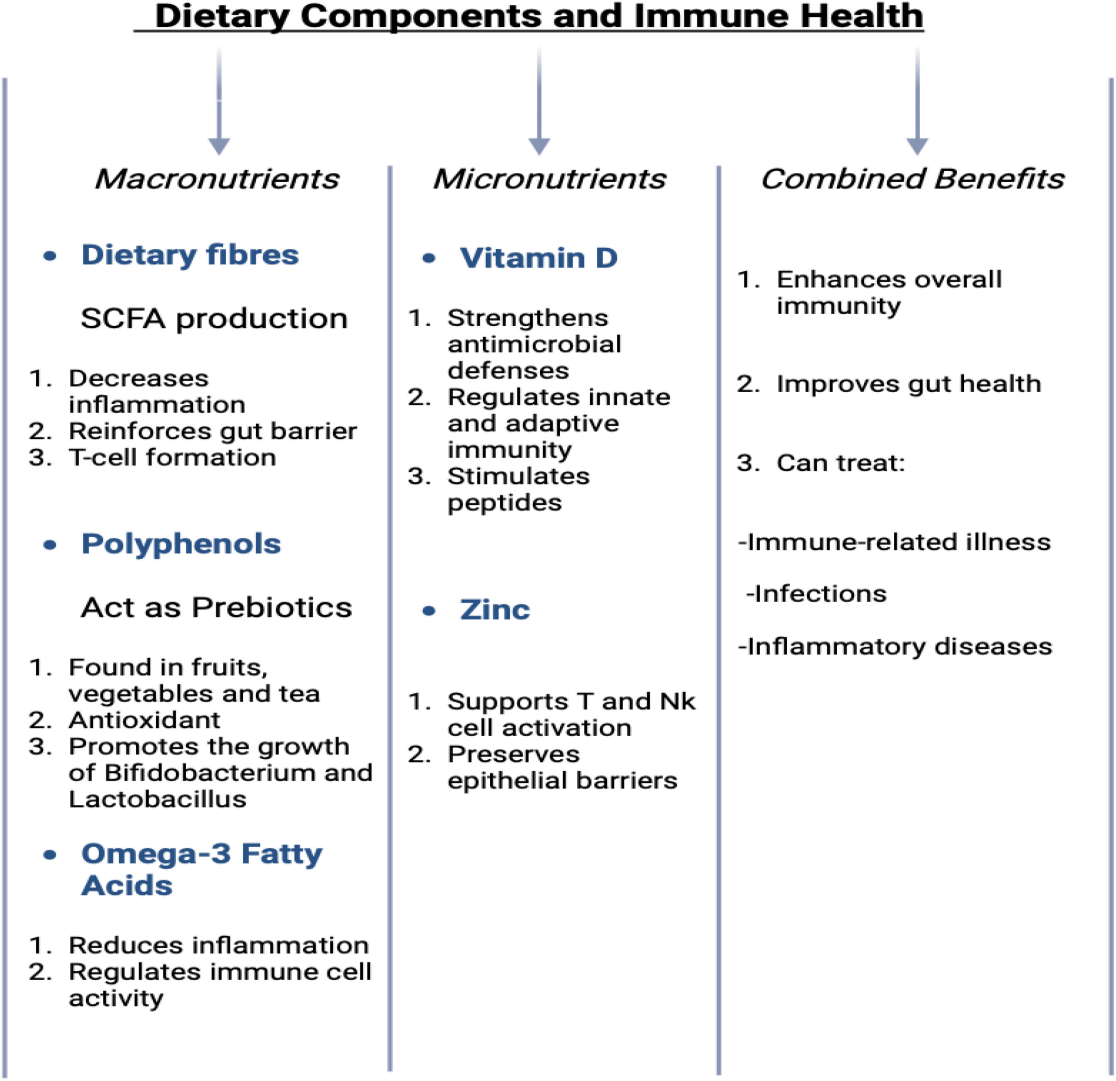
Role of various dietary components in human’s immunological health.

Dietary components provide not only essential nutrients for the body’s physiological processes but also substrates that nourish the microbiota within the gastrointestinal tract, collectively known as the gut microbiome (139). This symbiotic relationship between the host and the microbiome is critical for maintaining health **Table 5**. Key dietary elements such as dietary fibers (140), polyphenols, and fatty acids play significant roles in modulating the gut microbiota and supporting immune function. Their combined effects are essential in maintaining a balanced immune system and improving overall health, as demonstrated in (**Figure 4**) (141). Gut bacteria metabolize dietary fibers into SCFAs, including butyrate, which contribute to the reinforcement of the intestinal epithelial barrier, reduce inflammatory responses, and promote the differentiation and activity of regulatory T cells (142). These SCFAs are essential for maintaining gut health and preventing dysregulated immune responses. Some of the compounds such as polyphenols, found abundantly in fruits, vegetables, and beverages such as tea, act as prebiotics. These compounds possess potent antioxidant and anti-inflammatory properties and facilitate the growth of beneficial gut bacteria, including *Lactobacillus* and *Bifidobacterium* (143). These bacteria, in turn, help maintain a balanced microbial ecosystem and modulate immune responses, thereby supporting systemic health. Additionally, omega-3 fatty acids, which are present in fatty fish and certain plant oils, exert anti-inflammatory effects and modulate immune cell activity, further contributing to the resolution of chronic inflammation and the maintenance of immune homeostasis (144).

Dietary interventions are crucial in managing various inflammatory diseases, including neuroinflammatory, gastrointestinal, autoimmune, and cardiovascular diseases. In neuroinflammatory conditions, dietary fibers, polyphenols, and omega-3 fatty acids help modulate the gut microbiota, reduce inflammation, and improve immune function, with clinical evidence suggesting benefits in conditions like Alzheimer’s and multiple sclerosis (145). For gastrointestinal diseases like Crohn’s and ulcerative colitis, high-fiber diets and polyphenols enhance gut barrier integrity and reduce inflammation, showing promise in clinical trials (146). In autoimmune diseases such as rheumatoid arthritis and lupus, omega-3 fatty acids and polyphenols have demonstrated significant anti-inflammatory effects, improving symptoms and immune regulation (147). Furthermore, in cardiovascular diseases, diets rich in omega-3s and antioxidants have been shown to reduce oxidative stress.

By supporting both the gut microbiota and immune function, these nutrients provide a holistic approach to managing inflammation and immune dysregulation. In animal models, the ketogenic diet led to reduced systemic inflammation, improved brain function, and decreased levels of pro-inflammatory cytokines in the central nervous system. Furthermore, the diet modulated immune cell populations, particularly by reducing the activation of microglia, the resident immune cells in the brain. These findings suggest that dietary interventions can modulate the gut-brain immune axis and provide therapeutic benefits in conditions that involve neuro-inflammation (21). Tryptophan, an essential amino acid, plays a critical role in maintaining normal growth, health, and metabolic functions in both humans and animals. Beyond its fundamental physiological functions, tryptophan has a significant impact on modulating the gut-brain axis (GBA) through the metabolites produced during its metabolism by gut microbiota. Microbial communities in the gut can convert tryptophan into a range of bioactive compounds, most notably indole and its derivatives. These metabolites have profound effects on immune regulation and the maintenance of gut homeostasis (148). Indole derivatives, in particular, interact with the aryl hydrocarbon receptor a ligand-activated transcription factor present on immune and epithelial cells. Activation of AhR by these metabolites influences various immune cell functions, including the differentiation and expansion of regulatory T cells (Tregs), which are critical for maintaining immune tolerance and controlling inflammatory responses (149). This interaction not only influences immune cell activity but also modulates cytokine production, promoting an anti-inflammatory profile. By facilitating the differentiation of Tregs, indole derivatives help regulate local gut immunity and systemic immune responses, thus balancing the production of pro-inflammatory and anti-inflammatory signals. Additionally, these metabolites play a crucial role in gut barrier function, enhancing epithelial cell integrity and strengthening the intestinal mucosal barrier. This contributes to preventing the translocation of pathogens and reducing intestinal permeability, further supporting immune function and gut health (149). The metabolic products of tryptophan metabolism thus serve as a key link between gut microbiota activity and immune system regulation. Through their interaction with AhR, indole derivatives connect microbiome function with immune responses, ultimately enhancing both gut health and systemic immunity. This integration underscores the importance of the gut microbiota in modulating immune processes and highlights the therapeutic potential of tryptophan-derived metabolites in managing gastrointestinal (150) and systemic inflammatory conditions (117).

### 6.4 Immunomodulation via microbiome-derived metabolites in cancer therapy

A growing area of research is the use of microbiome-derived metabolites to modulate the immune system in the context of cancer therapy (**Table 6**).

**Table 6:** The role of dietary and microbiome-derived components in gut microbiota modulation and immune function.

Mechanism	Role in ICI Efficacy	Key References
SCFA Production & Treg Modulation	*Faecalibacterium prausnitzii* produces butyrate, which promotes regulatory T cell (Treg) differentiation while enhancing CD8+ T cell activation in the tumor microenvironment. This reduces inflammation and boosts anti-tumor immunity.	(151)
Increased Antigen Presentation	Firmicutes enhance dendritic cell maturation and MHC class I expression, improving tumor antigen presentation and activation of cytotoxic T cells.	(152)
PD-1/PD-L1 Pathway Modulation	Higher levels of *Firmicutes* correlate with increased CD8+ T cell infiltration, enhancing PD-1 blockade efficacy.	(8)
Gut Barrier Integrity & Systemic Immunity	*Firmicutes* upregulate tight junction proteins (e.g., occludin, ZO-1), preventing microbial translocation and systemic inflammation, which could otherwise suppress anti-tumor responses.	(153)

Recent evidence suggests that the gut microbiome plays a crucial role in influencing responses to immune checkpoint inhibitors (ICIs), such as anti-PD-1 and anti-CTLA-4 therapies, which are widely used in cancer treatment. Among the microbial taxa associated with enhanced ICI efficacy, Firmicutes, particularly *Faecalibacterium prausnitzii*, *Ruminococcus* spp., and *Clostridium cluster XIVa*, have been identified as key contributors to a favorable immunological landscape (154).

Mechanistically, Firmicutes influence ICI efficacy through several pathways. *Faecalibacterium prausnitzii* produces butyrate, a short-chain fatty acid that plays a crucial role in regulatory T cell (Treg) differentiation while simultaneously supporting CD8+ T cell activation in the tumor microenvironment. This dual effect reduces chronic inflammation while enhancing anti-tumor immunity (23). Additionally, certain Firmicutes species promote dendritic cell maturation and MHC class I expression, which in turn enhances tumor antigen presentation and activation of cytotoxic T cells (155). Higher levels of *Firmicutes* have been correlated with increased infiltration of cytotoxic CD8+ T cells, which improves the effectiveness of PD-1 blockade therapies (156). Furthermore, *Firmicutes* reinforce gut barrier integrity by upregulating tight junction proteins such as occludin and ZO-1, thereby preventing microbial translocation and systemic inflammation, both of which are known to suppress anti-tumor responses (22, 157, 158).

Clinical studies have provided compelling evidence supporting the association between Firmicutes and ICI response. A landmark study by Routy et al. (8) demonstrated that cancer patients with a higher abundance of *Firmicutes* in their gut microbiome had improved progression-free survival and overall response rates to anti-PD-1 therapy. Similarly, Gopalakrishnan et al. (159) found that melanoma patients who responded well to ICIs had increased levels of *Firmicutes*, particularly *Ruminococcus* and *Faecalibacterium*, compared to non-responders. Fecal microbiota transplantation (FMT) studies further validated this association, as transplanting microbiota from ICI responders into germ-free mice restored anti-tumor immunity and enhanced PD-1 blockade efficacy (8).

These findings underscore the potential for modulating the gut microbiota through probiotics, dietary interventions, or FMT as a strategy to optimize ICI therapy outcomes. Future research should focus on identifying specific microbial signatures that could serve as predictive biomarkers for immunotherapy response and exploring the potential of microbiome-targeted interventions in cancer treatment (160).

## 7 Challenges and ethical considerations

Despite the promising therapeutic potential of fecal microbiota transplantation (FMT), several significant challenges remain that must be addressed before its widespread adoption in clinical practice (161). One of the primary issues is the variability in outcomes, which can arise from differences in the donor microbiota as well as the methods used in stool preparation and administration. These variations can result in inconsistent therapeutic effects, making it difficult to predict patient responses (95). Standardizing the entire FMT process, including donor screening, stool preparation, and administration techniques, is essential to ensure reliable and safe results (162). Fecal microbiota transplantation: An update on clinical practice (162) This standardization would help to minimize variability and improve the reproducibility of FMT’s outcomes (132). Additionally, stringent donor screening is essential to prevent the transmission of infectious agents, antibiotic-resistant bacteria, or other undesirable genetic material through FMT, which could pose serious health risks to recipients (163, 164).

Similarly, while probiotics and prebiotics have demonstrated significant immunomodulatory and gut health benefits, their efficacy and stability remain key concerns. The viability of probiotics is influenced by factors such as storage conditions, gastrointestinal survival, and their ability to colonize the gut effectively. Many probiotic strains exhibit strain-specific effects, meaning their therapeutic potential is not always universally applicable (128). Additionally, the stability of probiotics in commercial formulations often poses challenges, as exposure to heat, moisture, or gastric acidity can reduce their potency before they reach the intestine (127). Prebiotics, on the other hand, rely on selective fermentation by beneficial gut microbes to exert their effects, but individual variations in gut microbiota composition can impact their effectiveness, leading to differential immune and metabolic responses among individuals (119).

Beyond efficacy, long-term clinical studies are needed to assess the safety and durability of FMT, probiotics, and prebiotics. While these interventions show promise in managing conditions like inflammatory bowel disease, allergies, and metabolic disorders, their long-term impact on the host microbiome remains uncertain. Potential risks include microbiome imbalances, unintended immune reactions, or the emergence of dysbiosis-related diseases over time (134). Addressing these challenges through rigorous research, improved formulation technologies, and personalized approaches will be essential to fully harness the benefits of microbiome-based therapies while minimizing risks.

Furthermore, challenges include the standardization of personalized microbiome-based treatments and the need for long-term safety evaluations to ensure the stability and predictability of therapeutic outcomes. These factors must be considered to optimize the clinical translation of microbiome-based interventions.

## 8 Current status of research in this area

In recent decades, research on the host-microbe immunological axis has experienced significant growth, with studies emerging from diverse geographical regions worldwide (165).

Microbiome research has predominantly been led by North America, Europe, and Asia, regions with substantial funding and advanced research infrastructure. A study analyzing over 444,000 human microbiome samples found that more than 71% originated from Europe, the United States, and Canada, with the U.S. alone accounting for 46.8% (166). However, there is a growing interest in microbiome research from developing regions. In Africa, microbiome studies have been conducted in 61% of the countries, with South Africa, Kenya, and Uganda leading the efforts (167). This field has advanced considerably with the advent of cutting-edge technologies. The application of multi-omics techniques, such as transcriptomics, metabolomics, and metagenomics (168), has enabled comprehensive studies of microbiomes, revealing both microbial diversity and functional roles at unprecedented levels (169). Single-cell and spatial transcriptomics have provided detailed insights into the complex interactions between immune cells and microbiota (170). Additionally, machine learning and artificial intelligence (AI) have revolutionized data analysis by generating predictive models and uncovering microbial biomarkers, thus enhancing our understanding of microbial ecosystems and facilitating the development of personalized therapeutic interventions (171). The exploration of the complex relationships between immunity and the microbiota is crucial for advancing our knowledge of human health and disease. This endeavor employs a variety of methodological approaches, including in silico techniques, animal models, and next-generation *in vitro* systems, each of which has its own advantages and limitations (170). Animal models provide in-depth biological context but present ethical and logistical challenges. Modern *in vitro* systems offer solutions to these concerns by reducing animal use, though they come at a higher cost and require specialized materials. In silico methods, while offering potential for resource efficiency and sustainability, also need ongoing refinement and rigorous experimental validation to ensure their reliability (172). Together, these integrative approaches support a more holistic understanding of the microbiome-immunity axis, paving the way for future research that is both sustainable and ethically responsible (173).

## 9 Conclusion

The host-microbe immunological axis plays a crucial role in maintaining human health and regulating immune responses. Advances in technologies like machine learning, single-cell analysis, and multi-omics have provided deeper insights into this complex relationship, paving the way for novel therapeutic approaches. Interventions such as probiotics, prebiotics, and fecal microbiota transplantation show promise in treating immune-related disorders and microbial imbalances. However, challenges remain, including ethical concerns, technological standardization, and understanding the long-term effects of microbial therapies. Future research should focus on personalized treatments, address these challenges, and further explore the potential of microbial modulation in promoting human health.

## 10 Future directions

Future advancements in microbiome therapy will focus on individualized treatments tailored to each patient’s unique microbiome composition, driven by innovations in machine learning, sequencing technologies, and a deeper understanding of microbiome dynamics. This will include therapies such as fecal microbiota transplants, dietary interventions, prebiotics, probiotics, and Microbiome-derived therapeutics. However, challenges remain in addressing population variability, predicting outcomes in complex microbial ecosystems, and ensuring the affordability and accessibility of personalized treatments. Ethical concerns regarding informed consent, data privacy, and equitable access must be addressed, along with the development of standardized regulatory frameworks to ensure safety and efficacy. Moreover, ongoing research is needed to explore the stability of microbial-immune interactions, assess long-term effects, and evaluate potential risks, such as ecological shifts or resistance. By addressing these scientific, ethical, and regulatory hurdles, microbiome-based therapies have the potential to revolutionize human health and offer targeted interventions for immune-related disorders. These therapies could have a transformative impact on diseases with immune-related components, such as Inflammatory Bowel Disease (IBD), autoimmune diseases like rheumatoid arthritis, asthma and allergies, cancer, metabolic disorders (e.g., obesity and type 2 diabetes), infectious diseases (e.g., HIV and tuberculosis), chronic fatigue syndrome, and neurodegenerative diseases (e.g., Alzheimer’s and Parkinson’s). In these conditions, microbiome modulation could offer a novel approach to restore immune balance, reduce inflammation, enhance treatment efficacy, and provide long-term benefits to patients. A more detailed discussion on the challenges of microbiome-based therapies, including standardization, regulatory hurdles, and ethical concerns, is essential to fully elucidate the complexities of their clinical application and the role of AI and precision medicine in overcoming these barriers.
